# Could high serum neopterin levels serve as a biomarker for patients diagnosed with rosacea, specifically the papulopustular rosacea subtype?

**DOI:** 10.1515/med-2026-1435

**Published:** 2026-05-20

**Authors:** Kübranur Ünal, Mehmet Alp Sert, Name Cemşitoğlu, Çağrı Emin Şahin, Mehmet Emre Erol, Nilsel İlter

**Affiliations:** Department of Medical Biochemistry, Faculty of Medicine, Gazi University, Ankara, Türkiye; Department of Medical Biochemistry, Kutahya City Hospital, Kütahya, Türkiye; Department of Dermatology and Venereology, Acibadem Fulya Hospital, Istanbul, Türkiye; Epidemiology Program, Institute of Health Sciences, Istanbul Medipol University, İstanbul, Türkiye; Department of Medical Biochemistry, Institute of Health Sciences, Gazi University, Ankara, Türkiye; Department of Dermatology and Venereology, Faculty of Medicine, Gazi University, Ankara, Türkiye

**Keywords:** rosacea, neopterin, inflammation, papulopustular rosacea, TNF-α, IFN-γ

## Abstract

**Objectives:**

This study aimed to compare serum neopterin and other inflammatory marker levels between patients with rosacea and healthy controls and to investigate the potential role of neopterin in disease diagnosis and severity assessment.

**Methods:**

Forty drug-naive patients clinically diagnosed with papulopustular rosacea and forty healthy controls were included. The serum levels of neopterin and other inflammatory markers were measured. The Investigator’s Global Assessment (IGA) score was used to assess the patient’s disease severity.

**Results:**

Serum neopterin, TNF-α, IFN-γ, and CRP levels were significantly higher in patients with papulopustular rosacea than in controls. A positive correlation was found between neopterin, TNF-α, IFN-γ, and IGA scores (r=0.498, 0.523, and 0.432, respectively). ROC curve analysis demonstrated good diagnostic performance for IFN-γ (AUC=0.821), TNF-α (AUC=0.780), and neopterin (AUC=0.747).

**Conclusions:**

New biochemical markers are needed to assess rosacea severity. Neopterin may be a potential biomarker for rosacea.

## What is known?

It is known that inflammation plays a role in the aetiology of rosacea.

## What new information does this article contribute?

This study is the first to elucidate the diagnostic value of neopterin in patients with rosacea and its relationship to disease severity.

## Introduction

Rosacea is an inflammatory dermatosis that primarily affects the facial regions, including the cheeks, nose, and forehead, characterised by redness (erythema), telangiectasia, papules, and pustule-like lesions [1]. It comprises four clinical subtypes: erythematotelangiectatic, papulopustular, phymatous, and ocular rosacea [2]. The global prevalence is estimated to range between 3 % and 5 % [2], 3]. Rosacea remains a clinical and social problem today, since it can negatively influence self-esteem and quality of life due to its cosmetic importance [3]. The diagnosis of rosacea is primarily clinical; however, in atypical presentations or cases requiring differential diagnosis, histopathological evaluation through skin biopsy may be necessary [4].

Although the exact aetiology of rosacea has not been fully elucidated, infectious agents, genetic predisposition, and immune system dysregulation have been implicated. In addition to these factors, the inflammatory response is considered a primary cause of rosacea [5]. These ideas are supported by increased TLR-2 expression, associated with IL-1β release, in rosacea-like findings induced by LL-37, a proinflammatory peptide, as well as by the association of the HLA-DRA locus with both rosacea and inflammatory diseases [6]. More pronounced cellular inflammation was observed in the papulopustular type compared to the other subtypes; increased CD4+ T-cell infiltration and the influence of the KLK5 pathway, which mediates proinflammatory LL-37 activation, suggest that this subtype stands out in the immune-inflammatory context [7], 8]. However, further biochemical studies are still needed to clarify the role of inflammation in rosacea pathogenesis.

Neopterin is a molecule belonging to the pterin family (such as pteroylglutamic acid, tetrahydrobiopterin), synthesised from GTP (Guanosine triphosphate) [9]. Activated macrophages and monocytes primarily produce Neopterin in response to interferon-gamma (IFN-γ) stimulation. Since IFN-γ, synthesised by Th1 cells, increases neopterin synthesis, neopterin is an indirect indicator of the Th1-mediated immune pathway [10]. ELISA kits suitable for clinical use have been developed to measure neopterin levels, allowing accurate quantification [11]. Neopterin levels obtained from these measurements provide useful information regarding inflammatory and immunological conditions, such as assessing prognosis in infectious diseases (e.g., COVID-19, HIV), monitoring disease activity in autoimmune diseases (e.g., rheumatoid arthritis, systemic lupus erythematosus), and early detection of organ rejection in organ transplantation [12], [13], [14]. Therefore, neopterin should be considered a promising biomarker providing useful information in immune and inflammatory disease settings.

Given the inflammatory nature of papulopustular rosacea and the key role of Th1-driven immune activation, we aimed to evaluate serum neopterin levels in conjunction with selected inflammatory markers and to explore their relationship with clinical severity. We hypothesised that serum neopterin levels would be higher in treatment-naïve patients with papulopustular rosacea compared to healthy controls and would be positively correlated with disease severity. Accordingly, the research question was whether serum neopterin could serve as a potential biomarker reflecting disease activity in papulopustular rosacea. To our knowledge, this is the first study to evaluate serum neopterin levels in papulopustular rosacea.

## Materials and methods

### Study design

The study was designed as a prospective, cross-sectional, single-centre study. Demographic data, including age and gender, were recorded for all participants. Forty patients aged 18 years and older, clinically diagnosed with papulopustular rosacea, and 40 healthy control subjects were included. Participants were recruited from individuals who presented to the Dermatology Clinic of Gazi University Faculty of Medicine between December 2023 and December 2024. Serum samples were collected to compare the levels of CRP, WBC, IL-6, TNF-α, IFN-γ and neopterin between the papulopustular rosacea patient group and the healthy control group.

Exclusion criteria encompassed individuals under the age of 18, those diagnosed with rosacea subtypes other than papulopustular rosacea, individuals with a history of systemic and local medication use for rosacea or other dermatological conditions within the preceding four weeks, patients with intellectual disabilities or psychological disorders that hinder effective communication, those with a diagnosed malignancy, pregnant or breastfeeding individuals, and patients who declined treatment, follow-up, or study-related assessments. The only criterion for withdrawal from the study was the participant’s voluntary decision to discontinue participation.

### Clinical assessment

The Investigator’s Global Assessment (IGA) score was used to evaluate the patients. Based on the IGA scoring system, patients were assigned a score ranging from 0 to 4 following their clinical examination (0=clear, 1=almost clear, 2=mild, 3=moderate, 4=severe). Individuals in the healthy control group were defined as those with a clinical score of 0, while the patient group comprised individuals with scores of 2 or 3.

### Biochemical analysis

Consent was obtained from each participant to measure markers, and 10 mL of venous blood was collected from the antecubital vein into serum separator tubes. Venous blood samples were clotted for 30 min at room temperature. Then, the samples were centrifuged at 1.300 *g* for 10 min, and the separated serum was collected. Following centrifugation, serum samples were aliquoted and stored at −80 °C until the day of analysis. All samples were thawed only once and analysed on the same day using the same commercial assay kit, in accordance with the manufacturer’s protocols, to minimise inter-assay variability. Hemolysed and icteric serums were excluded. Laboratory parameters, including WBC count, IL-6, TNF-α, IFN-γ, and neopterin levels, were measured. Neopterin (Cat. No. E3155Hu) and IFN-γ (Cat. No. E0105Hu) were measured using commercial enzyme-linked immunosorbent assay (ELISA) kits according to the manufacturer’s instructions (BT LAB, China). TNF-α levels were measured using original commercial ELISA kits (DIAsource ImmunoAssays S.A., Belgium). IL-6 serum levels were reviewed using an electro-chemiluminescence immunoassay (Elecsys; Roche Diagnostics, Rotkreuz, Switzerland). The IMAGE 800 nephelometric analyser (Beckman Coulter Inc., USA) was used to measure CRP concentrations. The lower limits of detection for serum neopterin, IFN-γ, TNF-α, IL-6, and CRP were 0.061 nmol/L, 0.49 ng/mL, 0.7 pg/mL, 1.5 pg/mL, and 0.8 mg/dL, respectively. The manufacturers’ reference ranges were 4.6–12.4 pg/mL for TNF-α, <7 pg/mL for IL-6, and 0–5 mg/dL for CRP. The analytical measurement ranges of the ELISA kits were 0.1–38 nmol/L for neopterin and 1–400 ng/mL for IFN-γ. The intra-assay coefficient of variation (CV) was 6 %, and the inter-assay CV was 10 % for neopterin and IFN-γ.

### Statistical analysis

IBM SPSS Statistics for Windows, version 25.0 (IBM Corp., Armonk, NY, USA) was used for statistical analyses. Continuous variables were presented as medians with interquartile ranges. Categorical data were summarised as frequencies and percentages. Differences in demographic and laboratory parameters between the patient and control groups were assessed using the Mann–Whitney U test for continuous variables and the Chi-square test for categorical variables. For the three groups categorised by IGA Scores, comparisons were performed using Kruskal–Wallis tests, followed by pairwise Mann–Whitney U tests where appropriate. Spearman’s rank correlation was used to examine the relationships among age, laboratory parameters, and other variables. The groups’ CRP, TNF-α, IFN-γ and neopterin diagnostic performance was calculated using the ROC Curve Analysis. The Binary Logistic Regression Analysis was used to examine the strength of the variables TNF-α, neopterin, and gender in predicting the probability of having rosacea. Two evaluations were made to ensure the logistic model fits the data well: the Nagelkerke R^2^ and the Hosmer–Lemeshow test. 95 % confidence intervals were reported using the Odds ratios (Exp(B)). To evaluate whether the association between serum neopterin levels and papulopustular rosacea was confounded or modified by sex, a binary logistic regression model was constructed including sex, neopterin, and a sex × neopterin interaction term. In this model, the interaction between sex and neopterin was not statistically significant (p=0.684), indicating that the relationship between neopterin levels and rosacea did not differ between males and females. Neither sex nor neopterin alone reached statistical significance within the interaction model, suggesting that sex-related effects did not drive the observed increase in neopterin levels in rosacea patients. Receiver operating characteristic (ROC) curve analysis was performed to evaluate the diagnostic performance of the biomarkers. Sensitivity, specificity, area under the curve (AUC), and optimal cut-off values were calculated, and optimal thresholds were determined using the Youden index.

#### Ethical approval and informed consent

The study protocol was approved by the Ethics Committee of Gazi University (Approval No.: 059, Date: January 17, 2025) and conducted in accordance with the Declaration of Helsinki and its later amendments.

Written informed consent was obtained from all participants prior to inclusion in the study.

## Results

Overall, 80 patients were included. Of them, 40 were in the control group, and the other 40 were in the patient group. There was a significant difference in gender distribution between the two groups (p<0.001). The patient group consisted of more females (90 %), whereas the control group was predominantly male (84 %).

**Figure 1:**
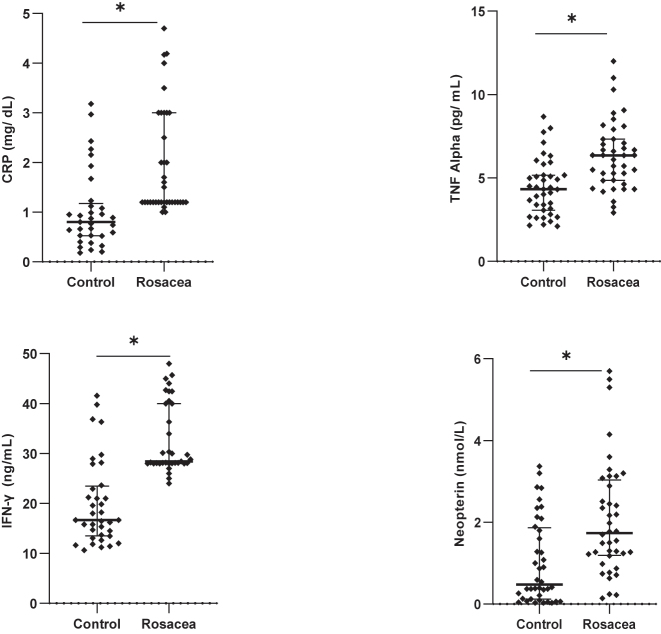
Comparison of serum CRP, TNF-α, neopterin and IFN-γ levels among study groups.

Although the median age was lower in the control group, the difference did not reach statistical significance (p=0.052). For laboratory parameters, C-reactive protein (CRP) levels in the patient group, the median was 1.20 mg/dL; interquartile range (IQR): 1.20–4.19 and for the control group, the median was 0.94 mg/dL; IQR: 0.55–2.39 (p=0.003), clearly indicating that the CRP levels were significantly elevated in the patient group. Similarly, TNF-α levels were more elevated in the patient group (median: 6.36 pg/mL; IQR: 4.85–7.33) compared to the control group (median: 4.32 pg/mL; IQR: 2.88–5.17) (p<0.001). Additionally, IFN-γ and neopterin levels were significantly elevated in the patient group (IFN-γ: median 29.93 ng/mL, IQR: 28.09–62.12; neopterin: median 1.74 nmol/L, IQR: 1.19–3.04) compared to the control group (IFN-γ: median 18.11 ng/mL, IQR: 13.88–28.75; neopterin: median 0.48 nmol/L, IQR: 0.12–1.87) (p<0.001 for both). Erythema scores, assessed using the Investigator’s Global Assessment (IGA), showed that 31 patients had moderate erythema (Score 3) and 9 had mild erythema (Score 2). The control group did not exhibit erythema (Score 0). The demographic and laboratory characteristics of the patient and control groups are summarised in Table 1. Comparison of serum CRP, TNF-α, Neopterin, and IFN-γ Levels between the study groups is given in Figure 1.

**Table 1:** Comparison of demographic characteristics and biochemical parameters in patient and control groups.

	Patient group (n=40)	Control group (n=40)	p-Value
Gender (female)	36 (90 %)	19 (47.5 %)	<0.001
IGA (Score 2/Score 3)	9/31		
Age, Years	42.00 (25.5–51.5)	37.00 (22.25–48.0)	0.052
CRP, mg/dL	1.20 (1.20–4.19)	0.94 (0.55–2.39)	0.003
WBC, ×10^3^/µL	7.20 (6.0–7.78)	6.60 (5.73–7.60)	0.218
IL-6, pg/mL	4.43 (3.64–5.87)	5.61 (3.77–6.33)	0.229
TNF-α, pg/mL	6.36 (4.85–7.33)	4.32 (2.88–5.17)	<0.001
IFN-γ, ng/mL	29.93 (28.09–62.12)	18.11 (13.88–28.75)	<0.001
Neopterin, nmol/L	1.74 (1.19–3.04)	0.48 (0.12–1.87)	<0.001

Data are presented as median (IQR) or as a number (%). The Mann–Whitney U test was used.

The comparison of demographic and laboratory parameters among IGA Scores revealed significant differences in several variables (Table 2). Regarding gender distribution, the proportion of females was significantly higher in the IGA Score 3 group (83 %) compared to the IGA Score 2 group (17 %) and the control group (34.5 %) (p=0.030). Conversely, the control group had a higher proportion of males (84 %) than the IGA groups. For laboratory parameters, TNF-α levels were significantly higher in the IGA Score 3 group (median: 6.36 pg/mL; IQR: 5.28–8.10) compared to the control group (median: 4.32 pg/mL; IQR: 2.88–5.17) (p<0.001). Similarly, IFN-γ levels were significantly elevated in both IGA Score 2 (median: 33.95 ng/mL; IQR: 28.07–74.01) and Score 3 (median: 29.74 ng/mL; IQR: 28.08–44.02) groups compared to the control group (median: 18.11 ng/mL; IQR: 13.88–28.75) (p=0.047). Neopterin levels also showed a significant difference, being higher in the IGA Score 3 group (median: 1.98 nmol/L; IQR: 1.22–3.13) compared to the control group (median: 0.48 nmol/L; IQR: 0.12–1.87) (p=0.011). The neopterin levels of the IGA Score 2 group were also elevated (median: 1.50 nmol/L; IQR: 0.93–1.98). No significant differences were observed among the groups in terms of CRP (median: 1.20 mg/dL; IQR: 1.20–3.16 for IGA Score 2, 1.20–4.19 for IGA Score 3, and 0.55–2.39 for controls), WBC (median: 7.10 × 10^3^/µL; IQR: 5.65–7.45 for IGA Score 2, 7.40 × 10^3^/µL; IQR: 6.20–7.80 for IGA Score 3, and 6.60 × 10^3^/µL; IQR: 5.73–7.60 for controls), and IL-6 levels (median: 4.18 pg/mL; IQR: 3.66–5.10 for IGA Score 2, 4.59 pg/mL; IQR: 3.63–5.95 for IGA Score 3, and 5.61 pg/mL; IQR: 3.77–6.33 for controls) (p>0.05 for all).

**Table 2:** Examination of biochemical parameters in groups separated according to IGA scores.

	IGA score 2	IGA score 3	Control	p-Value
Age	26 (24–41.5)	44 (37–52)	37.00 (22.25–48.0)	0.055
CRP, mg/dL	1.20 (1.20–3.16)	1.20 (1.20–4.19)	0.94 (0.55–2.39)	0.702
WBC, 10^3^/µL	7.10 (5.65–7.45)	7.40 (6.20–7.80)	6.60 (5.73–7.60)	0.235
IL-6, pg/mL	4.18 (3.66–5.10)	4.59 (3.63–5.95)	5.61 (3.77–6.33)	0.444
TNF-α, pg/mL	5.28 (4.50–6.57)^a^	6.36 (5.28–8.10)^b^	4.32 (2.88–5.17)	<0.001
IFN-γ, ng/mL	33.95 (28.07–74.01)^a^	29.74 (28.08–44.02)^b^	18.11 (13.88–28.75)	0.047
Neopterin nmol/L	1.50 (0.93–1.98)^a^	1.98 (1.22–3.13)^b^	0.48 (0.12–1.87)	0.011

Data are median (IQR). The Kruskal–Wallis test was used, and the Mann–Whitney U test was used for post hoc analysis. ^a^A significant difference was found between the IGA, Score 2 group and the control group. ^b^A significant difference was found between the IGA, Score 3 group and the control group.

Correlation analysis revealed several significant associations between demographic and laboratory parameters (Table 3). Age showed positive correlations with CRP levels (ρ=0.300, p=0.007), WBC count (ρ=0.263, p=0.019), TNF-α levels (ρ=0.347, p=0.002), and neopterin levels (ρ=0.250, p=0.025), suggesting an age-related increase in selected inflammatory markers. CRP levels were positively correlated with WBC count (ρ=0.298, p=0.007), TNF-α levels (ρ=0.485, p<0.001), and IFN-γ levels (ρ=0.229, p=0.041), indicating a coordinated inflammatory response. TNF-α levels were also positively associated with IFN-γ levels (ρ=0.224, p=0.045). Neopterin levels demonstrated a positive correlation with age but did not show significant associations with other laboratory parameters. In contrast, IL-6 levels did not exhibit significant correlations with any of the variables analysed (p>0.05 for all).

**Table 3:** Correlation between biochemical parameter levels and IGA scores.

	Age	CRP, mg/dL	WBC, ×10^3^/µL	IL-6, pg/mL	TNF-α, pg/mL	IFN-γ, ng/mL	Neopterin, nmol/L
CRP, mg/dL	0.300^b^						
WBC, ×10^3^/µL	0.263^a^	0.298^b^					
IL-6, pg/mL	0.128	0.123	−0.013				
TNF-α, pg/mL	0.347^b^	0.485^b^	0.201	0.059			
IFN-γ, ng/mL	0.103	0.229^a^	−0.021	−0.164	0.224^a^		
Neopterin, nmol/L	0.250^a^	0.000	0.001	0.039	0.146	0.147	
IGA scores (0-2-3)	0.500^b^	0.333^b^	0.167	−0.103	0.498^b^	0.523^b^	0.432^b^

Correlation coefficients were calculated using Spearman’s rank correlation. ^a^p<0.05; ^b^p<0.01.

In a multivariable binary logistic regression model that included sex, TNF-α, and neopterin, female sex was independently associated with a higher odds of rosacea (B=1.840, p=0.010, OR=6.29; 95 % CI: 1.56–25.64). Higher TNF-α levels (B=0.592, p=0.001, OR=1.81; 95 % CI: 1.28–2.55) and higher neopterin levels (B=0.630, p=0.024, OR=1.88; 95 % CI: 1.09–3.25) were also associated with increased odds of rosacea. The model demonstrated good fit (Nagelkerke R^2^=0.55; Hosmer–Lemeshow test p=0.988).

1wTo evaluate whether the association between neopterin and rosacea was modified by sex, a sex × neopterin interaction term was added to the model; however, the interaction was not statistically significant (p=0.684), indicating that the association did not differ between males and females.

**Figure 2:**
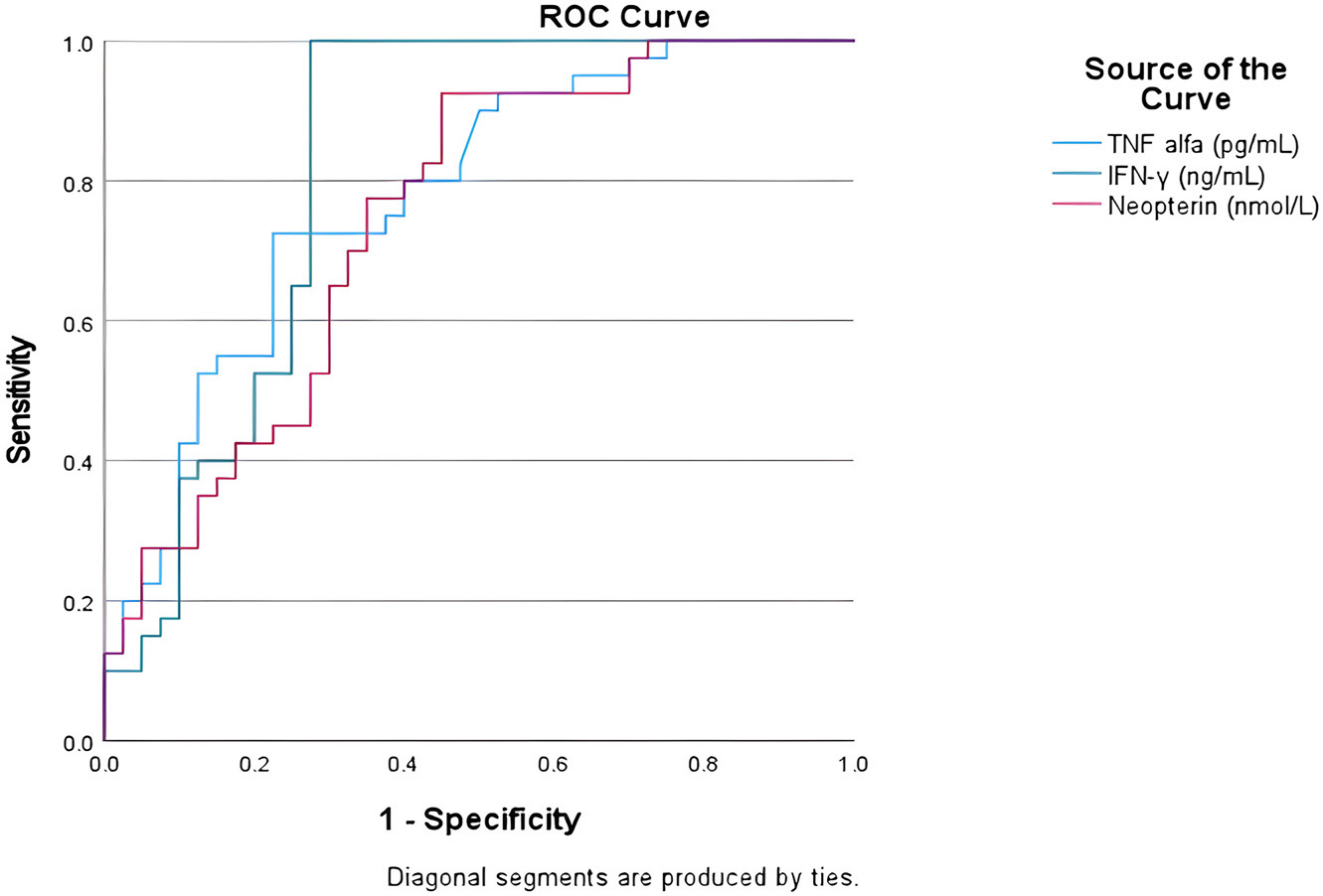
ROC curve of the TNF-α, neopterin and IFN-γ levels for the ability to differentiate the presence of papulopustular rosacea.

To evaluate the diagnostic performance of each biomarker, receiver operating characteristic (ROC) curves were generated. The results of this analysis are summarised in Table 4. IFN-γ showed the highest AUC value (AUC=0.821; p<0.0001) with a threshold of 27.99 ng/mL. IFN-γ is a strong potential diagnostic marker, demonstrating excellent sensitivity (97.5 %) and specificity (72.5 %). Similarly, TNF-α (pg/mL) showed a high AUC (AUC=0.780; p<0.0001) with a threshold of 4.15 pg/mL, a sensitivity of 92.5 %, and a specificity of 47.5 %. Neopterin (nmol/L) also demonstrated good diagnostic performance (AUC=0.747; p<0.0001), with a threshold of 0.61 nmol/L, a sensitivity of 92.5 %, and a specificity of 55 %. In contrast, in ROC analyses, IL-6 showed limited discriminatory performance, with low specificity at the selected cut-off, and therefore appears less suitable as a standalone diagnostic marker in this cohort. The diagnostic accuracy performance of TNF-α, neopterin, and IFN-γ is described in Table 4. Also, ROC curves were plotted (Figure 2).

**Table 4:** Diagnostic accuracy performance of TNF-α, neopterin and IFN-γ.

Parameters	AUC	Cut-off	Sensitivity	Specificity	p-Value	Youden index	95 % confidence interval	
							Lower bound	Upper bound
TNF-α, pg/mL	0.780	4.15	0.925	0.475	0.0001	0.400	0.679	0.880
IFN-γ, ng/mL	0.821	27.99	0.975	0.725	0.0001	0.700	0.720	0.921
Neopterin, nmol/L	0.747	0.61	0.925	0.550	0.0001	0.475	0.639	0.854

## Discussion

In the present study, we addressed whether serum neopterin could serve as a reliable biomarker for papulopustular rosacea. We found that serum neopterin levels were significantly elevated in treatment-naïve patients with papulopustular rosacea compared with healthy controls, and neopterin levels were positively associated with disease severity. We also observed higher levels of selected inflammatory markers, supporting a Th1-driven inflammatory profile in papulopustular rosacea. Cytokines play a critical role in the pathogenesis of rosacea, an inflammatory [15]. Therefore, in the present study, we measured serum levels of several inflammatory markers, including neopterin, CRP, WBC, IL-6, TNF-α, and IFN-γ, in patients with papulopustular rosacea and healthy controls. We found that neopterin levels were significantly elevated in the rosacea group, indicating the activation of the Th1-macrophage immune pathway in these patients.

In our study, 90 % of the rosacea patients were female, representing a statistically significant difference compared to the control group. This finding is consistent with previous reports and meta-analyses indicating that rosacea occurs more frequently in women [16], 17]. Hormonal and immunomodulatory factors, including estradiol-mediated pathways, have been proposed as potential contributors to this sex predominance [18], [19], [20]. Sex-related differences in the prevalence and clinical presentation of rosacea have been reported, raising concerns about potential confounding in biomarker-based analyses. In the present study, although the sex distribution differed between groups, sex-adjusted modelling and interaction testing demonstrated that the association between serum neopterin levels and rosacea was consistent across sexes. The absence of a significant sex × neopterin interaction suggests that neopterin elevation reflects disease-related immune activation rather than a sex-specific biological effect. Notably, the inclusion of an interaction term allowed us to distinguish between confounding and effect modification. While confounding would imply a spurious association driven by sex distribution, effect modification would indicate sex-specific differences in the relationship between the biomarker and disease. Our findings support neither scenario, reinforcing the robustness of neopterin as a potential inflammatory biomarker in papulopustular rosacea independent of sex.

There was a significant increase in CRP levels in the rosacea group, consistent with studies evaluating systemic inflammation in rosacea patients [19], 21], 22]. On the other hand, there was no significant association between CRP levels and disease severity, consistent with the results of Karaosmanoğlu et al. [21] and Ertekin et al. [19]. However, a study published by Koç et al. [22] reported a positive correlation. Further research is needed to clarify the relationship between rosacea severity and CRP levels. As in Karaosmanoğlu et al., there was no significant difference in WBC levels between the rosacea and control groups [21]. Mechanistically, continuously elevated TNF-α can suppress hematopoiesis by hindering the proliferation of hematopoietic stem and progenitor cells, even while IL-6 promotes myeloid cell proliferation and maturation [23], 24]. In the study, WBC levels remained normal despite elevated CRP levels, suggesting cytokine-mediated suppression of hematopoiesis, as evidenced by significantly higher TNF-α levels in the rosacea group.

Neopterin, TNF-α, and IFN-γ levels were all significantly higher in patients with rosacea. Increased IFN-γ expression in rosacea lesions has been repeatedly demonstrated through tissue immunostaining studies [25]. Similarly, Gao et al. [26] and Jiang et al. [27] demonstrated increased TNF-α levels in serum and tissue samples from rosacea patients. We also observe increased M1 macrophage infiltration relative to M2 macrophages in rosacea lesions, further supporting active Th1 immune signalling [28]. Another significant finding is that M1 macrophages, upon IFN-γ stimulation, produce neopterin [29], indicating that elevated neopterin levels are a marker of Th1 pathway activation.

Experimental data have also shown that neopterin may limit inflammatory cell adhesion, mainly by suppressing the expression of adhesion molecules such as ICAM-1, VCAM-1, and MCP-1 [30]. On the other hand, LL-37 has been shown to have the opposite effect, increasing the expression of ICAM-1 and VCAM-1. Immunohistochemical studies in rosacea biopsies have confirmed these findings [31]. Additionally, neopterin has anti-inflammatory effects by promoting M2 macrophage polarisation rather than M1 macrophage polarisation [30]. Considering these findings, the elevated neopterin in rosacea suggests that both immune activation and a possible compensatory anti-inflammatory effect are occurring in these cells. However, further *in vitro* and *in vivo* studies are needed to validate these findings. As no previous research has examined neopterin levels in rosacea, our findings represent an original contribution, elucidating a potential neopterin-mediated immune response in its pathogenesis.

In our study, IL-6 levels did not differ significantly between patients with rosacea and the control group. The literature presents inconsistent findings regarding IL-6 in rosacea. While a study by Yang et al. [32] found no significant difference in IL-6 levels among rosacea patients, consistent with our findings, the opposite was observed in studies by Ertekin et al. [19] and Aygar et al. In contrast, Salamon et al. [33] reported lower IL-6 levels in patients with rosacea. These discrepancies may reflect the heterogeneity among rosacea subtypes, each characterised by distinct pathogenic mechanisms. For instance, neurovascular inflammation mediated by active transient receptor potential (TRP) channels is observed in the erythematotelangiectatic subtype of rosacea. However, activation of both innate and adaptive immune responses is observed in the papulopustular subtype [34], 35]. Prior studies assessing IL-6 have included multiple rosacea subtypes, which explains the inconsistent results across studies. Consequently, IL-6 stimulates IL-4 production and promotes Th2 differentiation, inhibiting Th1 polarisation. Th2 also enhances SOCS1 expression and disrupts IFN-γ signalling subsequently [36]. Hence, lower IL-6 activity in our cohort may reflect the dominance of Th1-driven immune responses.

Neopterin, IFN-γ, and TNF-α levels differed significantly among the IGA-2, IGA-3, and control groups, with the highest levels observed in the IGA-3 group and the lowest in the controls. Following treatment of rosacea patients, a recent study has found a significant decrease in IGA scores, TNF-α, and M1 Macrophage activity, suggesting that these biomarkers are associated with disease severity [26]. Moreover, sex, neopterin, and TNF-α have been identified as independent predictors of rosacea presence with binary logistic regression. This result reinforces our hypothesis that inflammation and Th1 pathway activation play integral roles in the pathophysiology of rosacea. The diagnostic performance of neopterin, IFN-γ, and TNF-α was also evaluated. The sensitivities were calculated using the optimal cut-off value and were 92.5 %, 97.5 %, and 92.5 %, respectively. The specificities were 55 %, 72.5 %, and 47.5 %, respectively.

The present study has several noteworthy strengths. To the best of our knowledge, this is the first study to investigate serum neopterin levels in patients with papulopustular rosacea, providing novel evidence for the role of the Th1-macrophage immune pathway in the disease’s pathogenesis. Additionally, including treatment-naïve patients eliminated the potential confounding effects of topical or systemic therapies on inflammatory markers. Furthermore, the use of rigorous statistical methods, including sex-adjusted logistic regression and interaction testing, strengthens the validity of our findings regarding neopterin as a biomarker.

However, certain limitations must be considered. First, the sample size was limited to a single clinical centre, which may affect the generalizability of the results. Second, the cross-sectional design precludes any definitive conclusions regarding the causal relationship between neopterin elevation and rosacea progression. Most importantly, while we statistically accounted for demographic differences, the substantial gender disparity between the patient and control groups (90 % female in the rosacea group) may still carry a risk of residual confounding. This imbalance should be taken into account when interpreting the diagnostic performance of the biomarkers. Future molecular studies are warranted to investigate the pathophysiology of rosacea further. By identifying and combining relevant biomarkers like these, we can develop new diagnostic tools with enhanced sensitivity and specificity, thereby reducing the need for invasive biopsies in cases that are clinically ambiguous.

## Conclusions

This study demonstrated that serum neopterin, TNF-α, and IFN-γ levels are significantly higher in patients with papulopustular rosacea, suggesting that the Th1-macrophage immune pathway is actively involved in rosacea pathogenesis. Furthermore, considering the positive correlation between neopterin levels and disease severity, neopterin could be a potential biomarker for rosacea. After reviewing the current literature, neopterin levels have not been previously studied in rosacea. Therefore, our findings provide novel insights into the pathophysiology of rosacea concerning the role of neopterin-mediated immune activation.

We encourage further large-scale and prospective studies to better understand the roles of cytokines in rosacea. To better understand the heterogeneous nature of the disease, further studies are needed to compare cytokine profiles across rosacea subtypes. With more longitudinal studies, we can better evaluate the dynamic changes in neopterin levels across disease stages. In-depth research about the different treatment responses could establish neopterin as a promising biomarker for future monitoring of rosacea activity and therapeutic efficacy.
